# Designing Sensitivity: A Comparative Analysis of Microelectrode Topologies for Electrochemical Oxygen Sensing in Biomedical Applications

**DOI:** 10.3390/mi13010141

**Published:** 2022-01-17

**Authors:** Daniel T. Bacheschi, Evan Z. Strittmatter, Sonya Sawtelle, Mohsen Nami

**Affiliations:** 1 Department of Electrical Engineering, School of Engineering and Applied Sciences, Yale University, New Haven, CT 06511, USA; daniel.bacheschi@yale.edu (D.T.B.); evan.strittmatter@yale.edu (E.Z.S.); sdsawtelle@gmail.com (S.S.); 2 Department of Neurosurgery, School of Medicine, Yale University, New Haven, CT 06511, USA

**Keywords:** CMOS compatible, solid-state oxygen sensing, ultramicroelectrode

## Abstract

The monitoring of dissolved oxygen is a key parameter in many fields, namely the treatment and monitoring of various cerebral traumas. Leveraging existing manufacturing techniques, electrochemical sensors hold the potential for compact, simple, and scalable dissolved oxygen sensors. Past studies have focused on the general design of such sensors, but a comparative study on the impact of microelectrode geometries for cerebral applications has been forthcoming. We present here the results of a characterization study conducted across solid-state sensors with varying microelectrode geometries. The electrode structures were covered with a Nafion membrane and included variations of the classic interdigitated microelectrode array in addition to a circular microelectrode array variation. Voltage sweeps were conducted while monitoring the devices’ sensing current responses across a 50.3 mmHg change in dissolved oxygen within a deionized aqueous solution. Half of the devices were identified as ultramicroelectrode designs that presented a greater dependence on electrode spacing and topology. The ultramicroelectrode-style (UME) interdigitated electrode (IDE) topology presented the greatest signal response at 25.24 nA/mmHg, an approximate eight-fold improvement in sensitivity from a non-UME variation with a sensitivity of 2.98 nA/mmHg. The design presented a linear response from 8.3 mmHg to 58.6 mmHg with r^2^ = 0.9743. The sensitivity improvement was attributed to the ultramicroelectrode structure’s amplifying diffusive feedback, which was enabled by the IDE topology and short electrode spacings.

## 1. Introduction

Throughout ecological, biomedical, and chemical applications, the close monitoring of dissolved oxygen levels may prove critical during process control, environmental condition monitoring, and clinical diagnostics. In industry, this is useful for quality control in water production, as well as sewage management [1]. For scientific applications in aquaculture, this is widely deployed to closely monitor aquatic systems that support various forms of aquatic life [2]. In clinical applications where reliability, accuracy, and biofunctionality are paramount, suitable dissolved oxygen sensors have proven immensely beneficial. 

One such clinical application is during neurological surgery for traumatic brain injuries (TBI). Damage to living brain tissue caused by an external mechanical force necessitates invasive forms of surgery during which the close monitoring of brain tissue oxygen levels is paramount for preventing tissue hypoxia [3]. Subsequent forms of “secondary injuries” that may develop as a consequence of TBI such as ischemia, hypoxia, seizures, and fever also have a direct impact on patient recoveries from various forms of cerebral injury. As such, it is critical to monitor patients’ cerebral status via parameters such as intracranial pressure, blood flow, and brain tissue oxygen. Compact, simple, and minimally invasive dissolved oxygen sensors are necessary for brain tissue health monitoring in order to prevent secondary brain injuries. 

Chemical sensing modalities such as titration and iodimetry measure dissolved oxygen levels with high accuracy, but require long processing times and delicate experimental setups [4]. Optical methods for oxygen detection such as photochemical fluorescence often require complex and costly experimental setups and technologies. In addition, photochemical probes are frequently unsuitable for minimally invasive medical applications due to their high rigidity and resulting lack of maneuverability. Finally, the use of lab instrumentation such as mass spectrometry and gas chromatography systems presents an accuracy–cost tradeoff with long processing times and the inability to perform real-time measurements [5]. Solid-state electrochemical sensing, on the other hand, offers a simple, powerful sensing modality with the possibility of real-time monitoring. In addition, the electrochemical modality is easily implemented at the micro-scale via complementary metal oxide semiconductor (CMOS) fabrication technologies, inheriting a mature manufacturing paradigm from the semiconductor industry. For these reasons, electrochemical sensing has emerged as a leading modality for the accurate, reliable, and low-cost monitoring of brain tissue oxygen levels.

The Clark-type electrode—a popular electrochemical sensor design—presents a tri-electrode system covered by a semi-permeable membrane and an electrolytic layer that exploits reduction–oxidation reactions to generate current readings that correspond to analyte concentrations. In the case of oxygen, the working electrode provides the reaction site for the reduction of dissolved oxygen with the surrounding water molecules [6]. The reference electrode follows a separate voltage biasing scheme and serves to stabilize the potential of the electrochemical system in solution [7,8]. Here, we present a solid-state Clark-type sensor that does not require an aqueous electrolyte layer between the membrane and electrodes [9].

The characterization of conventional sensors has been researched extensively using a wide variety of materials, and each has adopted various electrode designs [4,10,11]. Microdisks, interdigitated electrodes, and bar-like electrodes have all been implemented in the design of dissolved oxygen sensors for both environmental and medical applications [4,10,11,12]. A comparative study of different solid-state microelectrode topologies tailored specifically for operating in a cerebral context, however, is lacking. Typical brain tissue oxygen levels vary between 1 mmHg and 50 mmHg [13,14,15]. Given such constraints, a comparative study would enable improved sensor performance by informing designers about the advantages of certain electrode topologies for use in the brain. Here, we present such a comparative analysis, focusing on the performance of eight solid-state micro-scale electrode geometries covered with a biocompatible, conductive, oxygen-permeable membrane to characterize guiding design principles for cerebral tissue oxygen sensing.

## 2. Materials and Methods 

### 2.1. Electrochemical Sensing Principles

The solid-state electrochemical oxygen sensor detects dissolved oxygen by diffusing oxygen first through an oxygen-permeable membrane, then through a conductive polymer matrix. While Clark-style oxygen sensor setups that do not consume oxygen during detection have been demonstrated, this is not a required characteristic [16]. The sensor then drives the reduction of oxygen at the working electrode while driving its oxidation at the counter electrode. The electrode processes unfold according to the following redox reaction equations:

(1) $$2H_{2}O+O_{2}+4e^{-}\to 4OH^{-}\;(\mathrm{working}\;\mathrm{electrode})$$

(2) $$4OH^{-}\to 2H_{2}O+O_{2}+4e^{-}\;(\mathrm{counter}\;\mathrm{electrode})$$

**Figure 1:**
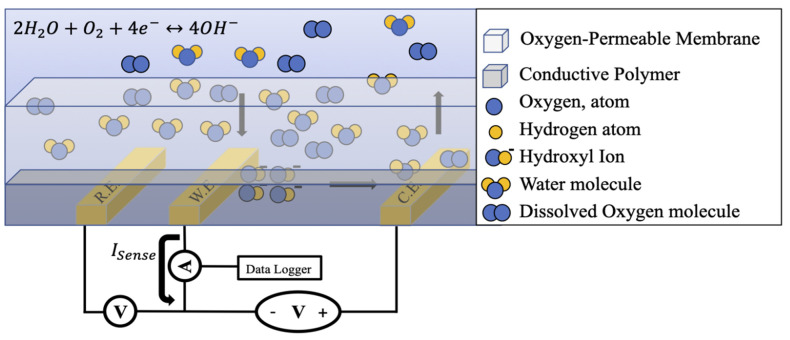
The working principle of a solid-state sensor geometry for dissolved oxygen electrochemical sensing. The reference and working electrodes are biased below the counter electrode in order to reduce oxygen and induce electronic charge transfer through the working electrode. The oxygen concentration is then captured by measuring the electron transfer rate as current.

The reduced oxygen reacts with water molecules to yield four hydroxyl ions, which then diffuse towards the counter electrode through the conductive polymer (Figure 1). Once ionic diffusion to the counter electrode is complete, oxidation occurs, and the hydroxyl ions decompose to yield dissolved oxygen and water. After decomposing, electrons are deposited into the counter electrode and are thereby returned to the sensing circuit.

Upon reduction, the working electrode donates four electrons to oxygen, generating a current proportional to the concentration of oxygen. The resulting charge transfer current is then described by the following equation:

(3) $$I_{sense}=\frac{nP_{effective}FA_{s}}{t_{m}}P_{O_{2}}$$

where *n* is the number of electrons exchanged between electrodes, $P_{effective}$ is the effective permeability of the selective membrane containing the influence of both lateral and vertical diffusion [8], *F* is Faraday’s constant, $A_{s}$ is the surface area of the working electrode, $t_{m}$ is the thickness of the selective membrane, and $P_{O_{2}}$ is oxygen’s partial pressure in solution. The effective permeability is dependent on the thickness of the selective membrane, the electrode size, and the electrode spacing.

With a strong linear dependence on effective permeability and electrode surface area, the electrode sensing configuration’s performance necessitates electrode design optimization. Kim et al. provided an analytical solution for the differential charge transfer current–membrane permeability relationship.

(4) $$\frac{\partial I_{sense}}{\partial P_{O_{2}}}=\left(\frac{nFA_{s}}{t_{m}}\right)P_{effective}\;$$

Electrochemical dissolved oxygen sensors employing sensing schemes depend significantly on the mass transport of molecular oxygen through the semi-permeable membrane and conductive polymer in order to generate a signal current proportional to the oxygen concentration. As a result, the protective, selective membrane isolating the sensing region must be permeable to dissolved oxygen. With this under consideration, it becomes clear that the diffusive mechanics of the movement of dissolved oxygen towards the working electrode are paramount for sensor operation. Such sensors effectively operate in one of two regimes, depending on the electrochemical properties of the sensing system. 

The over-voltage of a system is defined as being the difference between the thermodynamically predicted reduction potential of the redox reaction and the applied voltage bias during experimentation. When biased at low over-voltages, the sensor operates in the kinetic regime where the current response is highly dependent on the bias voltage applied across the counter and working electrodes [8,10]. After a transition voltage point, the rate of oxygen reduction at the working electrode matches the oxygen repletion rate via diffusion; from that point on, the sensor operates in the diffusion-limited regime [8,10]. As a result, the sensor current becomes linearly dependent on the concentration of dissolved oxygen in solution. The point of transition from the kinetic regime to the diffusion-limited regime is determined by the ratio of the diffusion-limited current to the charge transfer current occurring at electrochemical equilibrium, with smaller ratios leading to larger kinetic regime bias ranges [8,17]. 

On an additional note, the dissolved oxygen sensor’s versatility may be greatly expanded by tuning the membrane’s properties to better suit specific environments. Membrane biocompatibility, durability, and permeability may all be modulated to adapt the electrochemical sensor towards particular applications. 

### 2.2. Device Fabrication

**Figure 2:**
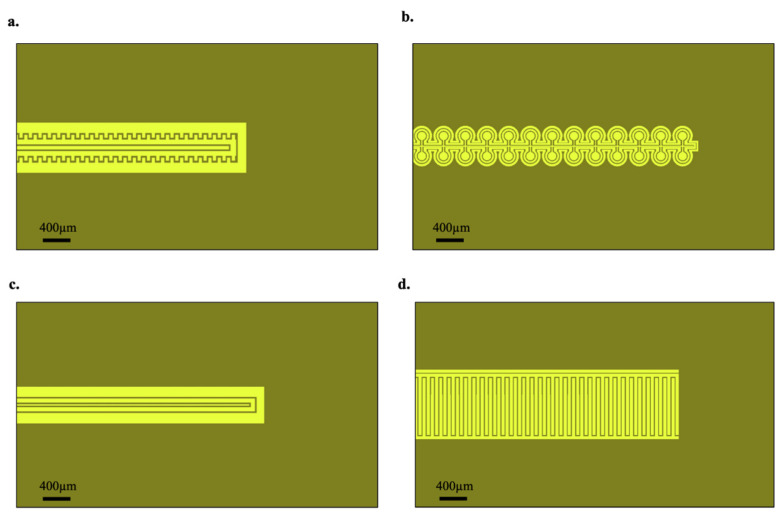
Sensor electrode geometries. (**a**) Electrode design designated as “serrated”, featuring a sawtooth-like counter electrode layout. (**b**) Electrode design designated as “omega” due to its working electrode design. (**c**) Electrode design designated as “basic”, featuring a simpler rectangular working electrode design. (**d**) Electrode design designated as “IDE” due to its interdigitated electrodes.

Devices were grown on a silicon wafer with 2 µm of thermal oxide on top, to mitigate potential electrode leakage paths. All electrodes were patterned via standard photolithography techniques using Shipley S1818 photoresist. The three electrodes were metallized with 10 nm of titanium covered by 100 nm of gold. The layout for each electrochemical sensor was standardized, with a 9 ${\mathrm{mm}}^{2}$ gold contact for each electrode, a 2.5 ${\mathrm{mm}}^{2}$ sensing window containing the electrodes, and the resulting lead lines from the window to the contacts. The variations in electrode geometry took place in the sensing window according to the pre-determined topologies (Figure 2). The total of 8 geometric variations were created by adjusting the width and spacing of certain features present in each of the 4 general electrode designs.

The oxygen-permeable, biocompatible Nafion membrane over the electrodes was formed via a dip-coating protocol resulting in thicknesses of 500 nm. An additional PDMS layer 10 µm in thickness was coated over the Nafion membrane in order to better protect the delicate Nafion from solution and thereby increase sensor longevity.

In order to arrive at an optimum microelectrode design, 4 different electrode topologies were fabricated (Figure 2). Variations of the widely-used interdigitated electrode (IDE), as well as a circular electrode geometry were tested, and their respective performances were measured with the changes of the dissolved oxygen concentration in solution.

### 2.3. Characterization Setup and Procedure

**Figure 3:**
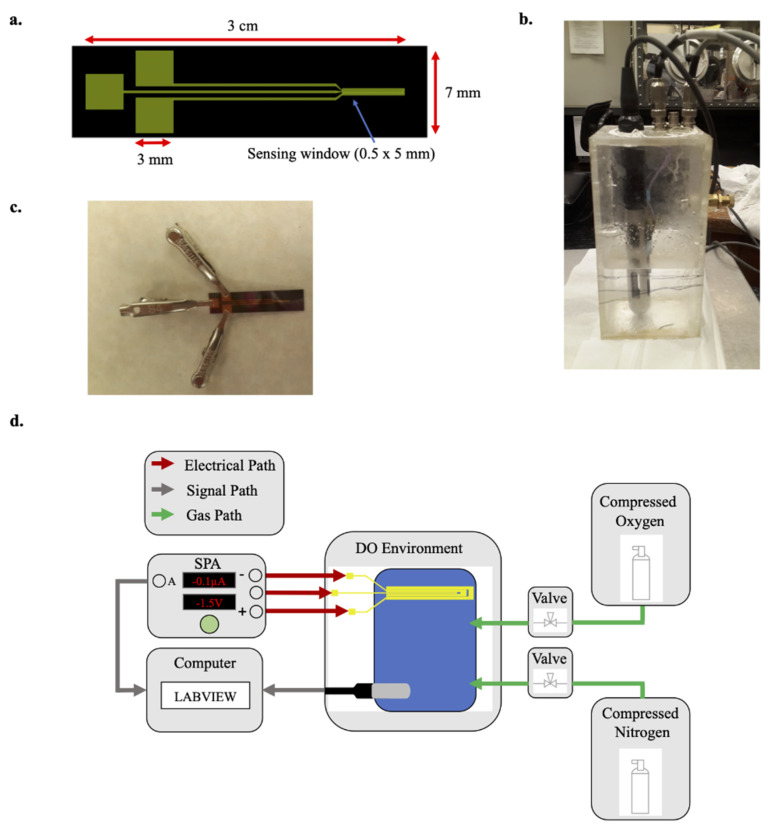
(**a**) CAD drawing of the standard overall electrochemical sensor geometry, featuring the sensing window and electrode contact pads. (**b**) Experimental setup, featuring the YSI ProDo Optical Probe in solution alongside the dissolved oxygen sensor. The container also had input ports for nitrogen and oxygen gas. (**c**) The sensor chip with metal leads attached for forming the biasing and sensing circuit with the SPA. (**d**) The overall electrical, signal, and gas paths throughout the experimental setup.

The electrochemical sensors were interfaced directly via a Semiconductor Parameter Analyzer machine (Agilent 4156B, Agilent Technologies, Santa Clara, CA, USA), which served as both the voltage bias source for driving oxygen reduction, as well as an ammeter for detecting sensing currents. All three of the sensor’s electrode pads were connected to the SPA via metal leads (Figure 3a,c). A container was filled with deionized water rich in dissolved oxygen, and both the electrochemical sensor’s electrodes and a commercial YSI ProDo Optical Probe were immersed in the water (Figure 3b). The ProDo Probe produced control measurements for the oxygen concentrations against which to evaluate the accuracy of the electrochemical sensor. The SPA conducted voltage sweeps from 0 V over-voltage to 1 V over-voltage across the working counter electrodes, with a voltmeter between the working and reference electrodes. Voltage sweeps were captured at 4 different oxygen concentrations: 8.3 mmHg, 25.1 mmHg, 41.6 mmHg, and 58.6 mmHg. The oxygen concentration was reduced by bubbling nitrogen gas into the water, with the nitrogen displacing the dissolved oxygen. A voltage sweep was conducted once the system reached steady-state after each step decrease in the oxygen concentration. Sensing current data from the SPA and the oxygen concentration data from the ProDo Probe were captured by a computer running LabVIEW for visual recording of the data. Figure 3d illustrates the overall experimental setup, with connections from the SPA driving the reduction–oxidation reactions, the oxygen concentration control circuit, as well as the signal data paths.

## 3. Results and Discussion

**Figure 4:**
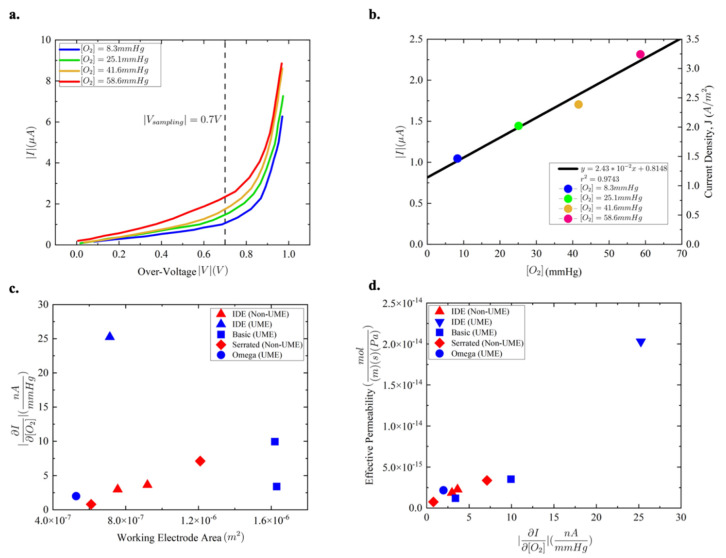
(**a**) I–V trace for the IDE_sep10 µm_width20 µm device, featuring multiple response signals across an over-voltage sweep from 0 V to −1 V at 4 chosen dissolved oxygen concentrations: 8.3 mmHg, 25.1 mmHg, 41.6 mmHg, and 58.6 mmHg. (**b**) Signal currents (µA) and current densities (A/m^2^) for the IDE_Sep10 µm_width20 µm device at the 4 different dissolved oxygen concentrations under a −0.7 V over-voltage device bias. (**c**) The device current response (nA/mmHg) according to a 50.3 mmHg change in oxygen concentration plotted against the working electrode area. A clear correlation with the working electrode area is present for the devices possessing standard microelectrode dimensions (red icons), whereas ultramicroelectrode devices (blue icons) appear to lose the correlation. (**d**) Device signal responses (nA/mmHg) plotted against the calculated permeabilities of the PDMS/Nafion membrane, displaying a clear positive correlation between signal response and permeability.

The sensor topologies were subjected to over-voltage sweeps from 0 V to −1 V across a range of oxygen concentrations. The changes in oxygen concentration were chosen to reflect the typical range of oxygen levels in cerebral tissues, from 1 mmHg to 50 mmHg [13,14,15]. The resulting I-V curves for the most responsive geometry configuration, the interdigitated geometry featuring finger widths of 20 µm and finger gaps of 10 µm in length, are presented (Figure 4a). In theory, one could operate the dissolved oxygen sensor in the diffusion regime under any bias above the necessary kinetic–diffusion regime transition point. At sufficiently high voltages, however, auxiliary redox reactions occur and supplement the sensing current. These reactions remove the sensor’s performance from a linear regime where dissolved oxygen concentration and sensor current are directly proportional. An exponential growth in signal current is observed past an over-voltage of −0.7 V (Figure 4a); for this reason, the standard biasing over-voltage was chosen to be −0.7 V for the devices’ analysis. 

A linear regression analysis revealed a strong, approximately linear relationship between the dissolved oxygen concentration and current response for the IDE_sep10 µm_width20 µm device (Figure 4b). A slope of 0.0243 µA per mmHg of dissolved oxygen was identified for the geometry’s current response. The regression analysis also yielded a strong $r^{2}$ value of 0.973, underscoring the high concentration dependence of the sensor’s response and confirming its operation in the diffusion-limited regime. In addition, the y-intercept suggests a baseline current of 0.8148 µA at the oxygen reduction potential for the IDE_sep10 µm_width20 µm design. The plotted current density in A/m^2^ was calculated by dividing the current regression by the working electrode area.

Figure 4c displays the working electrode area and experimental results for each geometry variation in the form of the change in current response in µA at a biasing over-voltage of −0.7 V across a 50.3 mmHg change in dissolved oxygen concentration. According to Equation (3), the device’s signal current response is linearly proportional to the working electrode area, as well as the concentration of the dissolved oxygen in solution. Several devices appear to deviate from the trend established by Equation (3), and at this point, it becomes necessary to introduce a separate class of microelectrode sensing structures: the ultramicroelectrode. Ultra-microelectrodes (UME’s) have been widely defined by the electrochemical community as being electrodes whose diffusion mechanics are completely radially dominated [18,19]. Upon entering the UME regime, the previous rules of thumb regarding electrode design no longer apply—e.g., increasing the working electrode area is no longer directly correlated with an increase in the signal response. While the standard microelectrode geometries holding all dimensions above 25 µm evince a clear working electrode area dependence, the UME geometries fail to abide by the same correlation (Figure 4c). 

**Figure 5:**
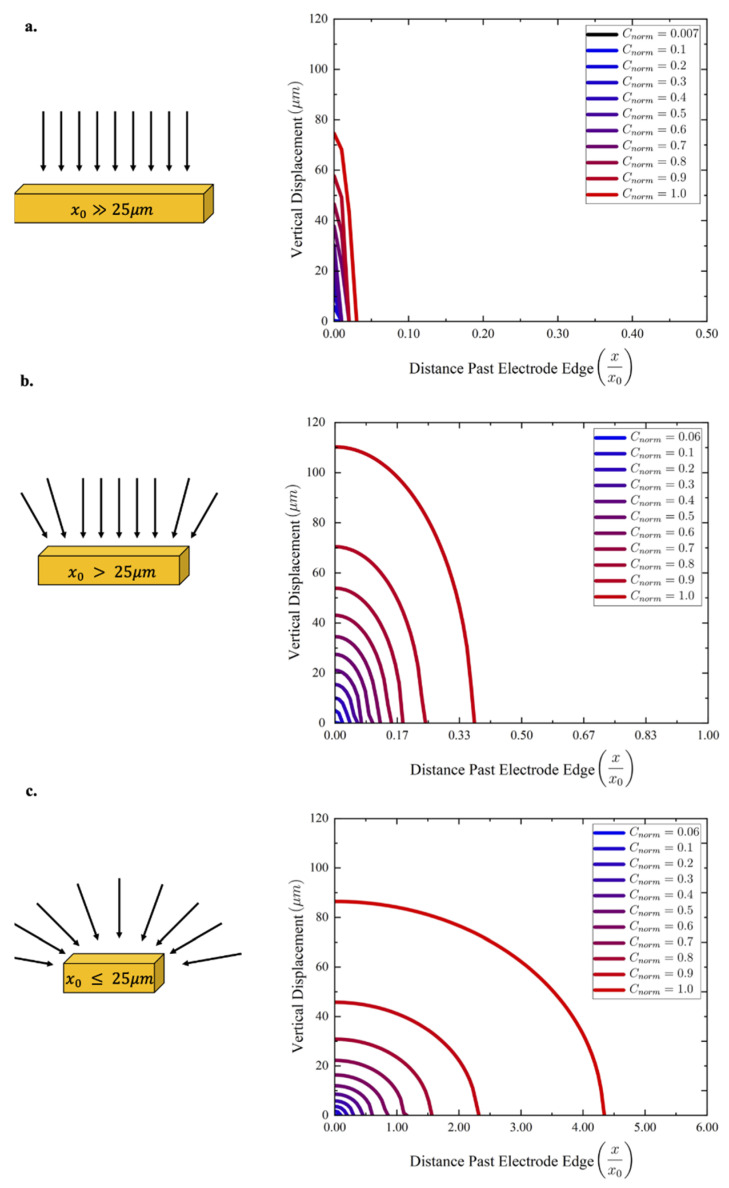
Normalized isoconcentration curves at different radial distances from the electrode surface, plotted along vertical versus horizontal displacement. Isoconcentration lines were plotted via Equation (5) after 1 s and are 0.1 normalized concentration units apart. The electrode edge is located at the origin of the isoconcentration plots. (**a**) (Left) The planar diffusion vectors for a microelectrode with a critical dimension of 3 mm. (Right) The isoconcentration lines, featuring a steep return to the bulk concentration upon leaving the electrode edge. (**b**) (Left) The semi-hemispherical diffusion vectors for a microelectrode with a critical dimension of 300 µm. (Right) The isoconcentration lines, featuring a delayed return to the bulk concentration shortly after leaving the electrode edge. (**c**) (Left) The radially dominated diffusion vectors for a microelectrode with a critical dimension of 20 µm. (Right) The isoconcentration lines, featuring a protracted return to the bulk concentration far from the electrode edge.

The primary reason for UME’s intriguing behavior is the role of edge effects in the diffusion kinetics of the system. When operating in a diffusion-limited regime, microelectrode systems’ diffusion kinetics are dominated by planar diffusion. Planar diffusion refers to a diffusion vector that is strictly orthogonal to the surface of the electrode, with an analyte concentration profile that returns to the bulk concentration outside the region directly above the electrode’s surface (Figure 5a). The surface of the electrode is assumed to be a point of zero concentration. The concentration as a function of radial distance from the electrode surface may be expressed analytically as:

(5) $$C\left(x,t\right)=C_{0}\left(1-\frac{x_{0}}{x}\mathrm{erf}\left\{\frac{\left(x-x_{0}\right)}{2{\left(Dt\right)}^{0.5}}\right\}\right)$$

where C(x,t) is the reduced analyte concentration at a radial distance x from the electrode surface at time t after reduction initiation, $C_{0}$ is the bulk concentration, $x_{0}$ is the characteristic dimension of the working electrode, and D is the diffusivity [20]. 

As at least one dimension of the microelectrode approaches the UME regime **(**Figure 5b), the diffusion takes on an additional radial component, extending the affected concentration profile beyond the electrode area [18,19,20]. Once the electrode enters the UME regime (Figure 5c), the radial diffusion component begins to dominate, and the concentration profiles become hemispherical, extending the effective diffusion region far beyond the surface of the microelectrode itself [18,19,20].

An increase in the effective concentration profile area gives rise to significant current densities despite low working electrode areas, all owing to a greater diffusion “collection area” [18,19,20]. These larger “collection areas” establish a higher sensitivity to changes in analyte concentration for ultramicroelectrode devices. While the total current may be lower than larger electrode designs, the change in signal current is much greater, boosting the robustness of microelectrode systems [17,19,20]. One critical repercussion of UME’s strong signal response is that the sum of sensing currents over an array of UME’s is larger than that of a single electrode of surface area equivalent to that of the UME array [17]. Consequently, when designing electrode-based electrochemical sensing systems, it is preferred to employ an array of UME’s over singular, larger electrodes.

Looking to the ultramicroelectrodes’ results in Table 1, the interdigitated electrode design presents a significant signal change of 1.27 µA in response to the 50.3 mmHg change in dissolved oxygen concentration. The concentration profile behavior described by Equation (5) and Figure 5 was determined to be one of the key reasons behind the design’s high sensitivity. The geometry’s larger concentration profile supported a far higher current response when compared to non-UME designs.

The second largest signal change from the UME device group was a 0.5 µA change from the Basic_sep10 µm_width20 µm geometry, nearly a factor half that of the IDE’s signal response. The interdigitated electrode array’s superior current response over other UME topologies indicates that the geometry itself presents an advantage over other UME designs. When the counter and working electrodes are sufficiently close together, the oxidized products at the counter electrode may diffuse back to the working electrode for re-reduction, establishing a diffusion feedback loop [19,21,22,23] Consequently, electrode separation plays a critical role in the electrochemical sensor design, as observed in the difference in sensor performance between the two basic designs (Table 1). Tripling the electrode separation length corresponded to a nearly proportional decrease in current response by a factor of 2.92, despite similar working and counter electrode areas.

While the diffusion feedback loop is characteristic of any electrode design with sufficiently small electrode spacing, the IDE geometry’s array of interdigitated working and counter electrodes poses an advantage in collection efficiency. As the dissolved oxygen is oxidized at one counter electrode, it diffuses to the next working electrode in the array and is then reduced again. In other words, the collection efficiency approaches unity for IDE designs [21,22,24,25]. Such a diffusion feedback loop leads to more robust and sensitive sensing, as even low changes in dissolved oxygen concentration will be amplified by the diffusion loop. When compared to designs of similar dimensions in the microelectrode group, the IDE’s amplification advantage becomes clear: the response from IDE_sep20 µm_width40 µm was larger than that of the Serrated_sep10 µm_width30 µm design by 375%, while presenting a 23% larger area. 

Using Equation (4), the effective permeability for each geometry was calculated and plotted against the geometry’s respective current response (Figure 4d). Owing to its large current response and radially dominated diffusion kinetics, the ultramicroelectrode IDE geometry presented the largest effective permeability. 

## 4. Conclusions

Dissolved oxygen concentration is a key parameter in monitoring processes for a wide array of industries and fields, and the optimized design of microelectrode-based oxygen sensors is critical for effective clinical applications. The analysis presented here investigated the sensing performance of eight variations of four microelectrode topologies against changes in dissolved oxygen in solution. Variations of the archetypal interdigitated electrode structure in addition to a circular electrode structure were investigated. In order to characterize the performance of each microelectrode geometry, the microelectrodes were covered with Nafion membranes, immersed in an aqueous solution containing dissolved oxygen, and subjected to voltage sweeps. Current data were collected, and the changes in steady-state, diffusion-limited current across a 50.3 mmHg change in oxygen concentrations were calculated for each geometry. The observed current responses agreed well with electrochemical electrode theory and pointed to the advantages ultramicroelectrodes pose over standard microelectrode designs. While the standard microelectrode designs’ current responses directly followed their working electrode areas, the ultramicroelectrodes presented a stronger dependence on electrode topology and the diffusion feedback loop established by tight electrode spacing. We showed that close to an eight-fold increase in sensitivity can be obtained using an ultramicroelectrode geometry. This study provides insight into the design of high-performance solid-state oxygen sensors for medical applications such as brain tissue oxygen monitoring. 

## Figures and Tables

**Table 1:** Current responses of the microelectrode and ultramicroelectrode geometries.

Geometry	Feature Width (µm)	Feature Separation (µm)	ΔI (µA)	$\Delta \left[O_{2}\right]\;(\mathrm{mmHg})$	$\mathrm{Working}\;\mathrm{Electrode}\;\mathrm{Area}\;(10^{-7}m^{2})$
Microelectrode Geometries					
Serrated	100	10	0.358	50.3	12.1
IDE	40	10	0.183	50.3	9.196
IDE	40	20	0.15	50.3	7.56
Serrated	30	10	0.04	50.3	6.1
Ultramicroelectrode Geometries					
IDE	20	10	1.27	50.3	7.13
Basic	20	10	0.5	50.3	16.2
Basic	20	30	0.171	50.3	16.3
Omega	20	15	0.1	50.3	5.27

## Data Availability

The data presented in this study are available upon request from the corresponding author. The data are not publicly available due to intellectual property and privacy concerns.
